# Heart Rate as a Marker of Relapse During Withdrawal of Therapy in Recovered Dilated Cardiomyopathy

**DOI:** 10.1016/j.jchf.2021.03.010

**Published:** 2021-07

**Authors:** Brian P. Halliday, Ali Vazir, Ruth Owen, John Gregson, Rebecca Wassall, Amrit S. Lota, Zohya Khalique, Upasana Tayal, Richard E. Jones, Daniel Hammersley, Antonis Pantazis, A. John Baksi, Stuart Rosen, Dudley J. Pennell, Martin R. Cowie, John G.F. Cleland, Sanjay K. Prasad

**Affiliations:** a Cardiovascular Research Centre, Royal Brompton Hospital and National Heart Lung Institute, Imperial College, London, United Kingdom; b Department of Medical Statistics, London School of Hygiene and Tropical Medicine, London, United Kingdom; c Robertson Centre for Biostatistics, University of Glasgow, Glasgow, United Kingdom

**Keywords:** cardiovascular magnetic resonance, dilated cardiomyopathy, recovered ejection fraction, relapse, ACE, angiotensin-converting enzyme, ARB, angiotensin receptor blocker, CMR, cardiovascular magnetic resonance, DCM, dilated cardiomyopathy, GLS, global longitudinal strain, LVEDV, left ventricular end-diastolic volume, LVEF, left ventricular ejection fraction, MRA, mineralocorticoid receptor antagonist, NT-proBNP, N-terminal pro B-type natriuretic peptide, *TTNtv*, truncating variant in the titin gene

## Abstract

**Objectives:**

The objective of this study was to determine the relationship between heart rate and relapse among patients in the TRED-HF (Therapy withdrawal in REcovered Dilated cardiomyopathy trial).

**Background:**

Understanding markers and mechanisms of relapse among patients with recovered dilated cardiomyopathy (DCM) may enable personalized management.

**Methods:**

The relationship between serial heart rate measurements and relapse was examined among patients in the TRED-HF trial, a randomized trial which examined the safety and feasibility of withdrawing heart failure therapy from 51 patients with recovered DCM over 6 months. In total, 25 patients were randomized to therapy withdrawal and 26 to continue therapy, of whom 25 subsequently began therapy withdrawal in a single arm crossover phase.

**Results:**

The mean ± SD heart rate for those who had therapy withdrawn and did not relapse was 64.6 ± 10.7 beats/min at baseline and 74.7 ± 10.4 beats/min at follow-up, compared to 68.3 ± 11.3 beats/min at baseline and 86.1 ± 11.8 beats/min at follow-up for those who relapsed. After adjusting for differences in heart rate at baseline, patients who had therapy withdrawn and relapsed had a 10.4 beats/min (95% CI: 4.0–16.8) greater rise in heart rate than patients who had therapy withdrawn and did not relapse (*P* = 0.002). After data were adjusted for age, log N-terminal pro–B-type natriuretic peptide, and left ventricular ejection fraction (LVEF), heart rate (per 10 beats/min; hazard ratio [HR]: 1.65; 95% CI: 1.10-2.57; *P* = 0.01) and change in heart rate from baseline (per 10 beats/min; HR: 1.70; 95% CI: 1.12–2.57; p = 0.01) were associated with relapse. The results remained qualitatively the same after adjusting for beta-blocker dose.

**Conclusions:**

For patients with DCM and improved LVEF, the rise in heart rate after treatment is withdrawn treatment identifies patients who are more likely to relapse. Whether the increase in heart rate is a marker or a mediator of relapse requires investigation. (Therapy withdrawal in REcovered Dilated cardiomyopathy trial [TRED]; NCT02859311)

Dilated cardiomyopathy (DCM) refers to a common phenotype that is the result of a diverse range of genetic and environmental triggers (1). A proportion of patients demonstrate recovery of both symptoms and left ventricular (LV) function (2). For some patients, this recovery may persist despite withdrawal of treatment. Other patients, however, relapse, indicating heart failure remission rather than sustained recovery or cure and the need for continuation of therapy (3). There are no reliable predictors to identify patients who will relapse after therapy is withdrawn. Therefore, patients with DCM whose ventricular function has improved are typically advised to remain taking disease-modifying treatments for heart failure lifelong (4).

Despite such recommendations, some patients remain interested in finding out if their medication can be stopped or reduced, accepting the risk of relapse. Clinicians may prefer to try to help their patients do this cautiously rather than let them experiment on their own. There may be specific situations, such as peripartum cardiomyopathy, where clinicians and patients agree to a trial of therapy withdrawal or dose reduction. The best way to monitor withdrawal or reduction of therapy to predict and prevent relapse is unknown (5,6).

Data from TRED-HF (Therapy withdrawal in Recovered DCM) demonstrated that deterioration in cardiac function, determined by cardiovascular magnetic resonance (CMR), often preceded a rise in plasma concentrations of N-terminal pro–B-type natriuretic peptide (NT-proBNP). This suggests that this biomarker may not be a good early marker of relapse. However, frequent follow-up using CMR is rarely feasible in clinical practice. Integrating readily available non-invasive variables that can be measured frequently, such as heart rate, with periodic assessment using advanced imaging techniques may be a feasible and robust monitoring strategy. A strong association among achieved heart rate, temporal changes in heart rate, and outcome among patients with heart failure is well established (7, 8, 9, 10). In the present study, the associations among change in heart rate and attained heart rate and the occurrence of relapse among patients in the TRED-HF trial was examined.

## Methods

The TRED-HF (NCT02859311) study was an open-label, randomized trial examining the safety and feasibility of phased withdrawal of heart failure therapy in patients recovered DCM. A comprehensive description of the methods is provided elsewhere (3). All patients provided informed consent. The study was approved by the London-Surrey Borders National Research Ethics Committee and authorized by the Medicine and Healthcare Products Regulatory Agency.

Briefly, 51 asymptomatic patients with a diagnosis of DCM with a prior left ventricular ejection fraction (LVEF) of <40% that had subsequently improved to ≥50% and who now had normal LV end diastolic volume (LVEDV) and an NT-proBNP concentration of <250 ng/l and who were still taking at least 1 heart failure therapy (eg, loop diuretic, beta-blocker, angiotensin-converting enzyme [ACE] inhibitor, angiotensin receptor blocker [ARB], or mineralocorticoid receptor antagonist [MRA]) were randomized 1:1 to phased withdrawal or continuation of pharmacological heart failure therapy for 6 months. At baseline, 16 weeks, and 6 months, all patients underwent comprehensive CMR assessment. After 6 months, patients assigned to the control arm subsequently entered a single-arm cross-over phase and had therapy withdrawn between months 6 and 12. Those patients were followed in the same way as the randomized phase of the study.

Therapy was withdrawn in a supervised, step-wise fashion over a maximum of 16 weeks. Changes were made every 2 weeks following clinic or telephone review. Heart rate, weight, and NT-proBNP concentrations were measured every 4 weeks with interim reviews taking place by telephone, provided patients remained asymptomatic. Loop diuretic drugs were withdrawn first, followed by MRAs, beta-blockers, and finally ACE inhibitors or ARBs. The medication was stopped if the participant was taking 40 mg or less of furosemide (or equivalent), 50 mg or less of spironolactone, or 25% or less of the recommended dose of beta-blocker or ACE inhibitor or ARB. If participants were prescribed larger doses of any of the above, the dosages were reduced by 50% every 2 weeks.

At each clinic visit, resting heart rate was recorded in beats/min by using a 12-lead electrocardiogram performed following 5 min of rest in the supine position. The primary endpoint was a relapse of DCM defined by any 1 of the following: 1) a reduction in LVEF by >10% and to <50%; or 2) an increase in LVEDV by >10% and to above the normal range; or 3) a two-fold rise in NT-proBNP from baseline and to >400 ng/l; or 4) clinical evidence of heart failure. Therapy was reintroduced as soon as any of the primary endpoint criteria were fulfilled. The management of patients who did not meet the primary endpoint but experienced adverse events was determined by the study team and the participant’s routine physicians.

### Statistical analysis

The main analyses were nonrandomized comparisons of heart rate measurements according to whether patients relapsed following therapy withdrawal. Accordingly, data from the therapy withdrawal arm were used during the randomized phase of TRED-HF (baseline at month 0, end of follow-up at 6 months) and from the control group during the cross-over phase (with baseline at month 6 and end of follow-up at month 12). As background, data from control patients are presented during the randomized phase of the trial when patients remained taking therapy, although these data were not used for formal statistical comparisons.

Three main analyses were performed. First, heart rate measurements at the end of the study or the time of relapse were compared between patients who did relapse and those who did not. Differences were estimated between these groups by fitting an analysis of covariance (ANCOVA) model with the heart rate at the end of the study (or relapse), 4 weeks and 8 weeks as the dependent variables and baseline heart rate and relapse status as the independent variables. Second, serial heart rate measurements taken at each study visit following therapy withdrawal was modeled separately according to whether or not a patient relapsed. To do this, fractional polynomial models with 4 knots were used. Third, the association between heart rates at each visit and relapses at either the same visit or until the time of the next heart rate measurement was estimated. To do that, Cox proportional hazards model was used, fitting heart rates at each visit as the time-updated covariate (11). Similar models were used to investigate the role of changes in heart rates (either from baseline or the previous visit). These models were adjusted for baseline heart rate. To examine whether heart rate may be able to predict future relapse, additional models investigated the association between heart rate variables at the preceding visit and relapses at the subsequent visit (Supplemental Appendix). We also performed several sensitivity analyses: an additional set of Cox models were adjusted for markers of disease severity at baseline: age, log NT-proBNP, and LVEF; to ensure that heart rate was not acting as a proxy for dose of beta-blocker, a further Cox model was also adjusted for beta-blocker dose as a time-updated variable. Finally, the correlation between change in heart rate and change in LVEF, global longitudinal strain (GLS) (as measured by feature tracking CMR) and NT-proBNP concentration between baseline and follow-up were examined using Spearman correlation coefficient.

A p value of <0.05 was taken as significant throughout. Statistical analyses were performed using Stata version 16.0 software (StatCorp). The investigators had complete access to all raw and derived datasets.

## Results

**Figure 1:**
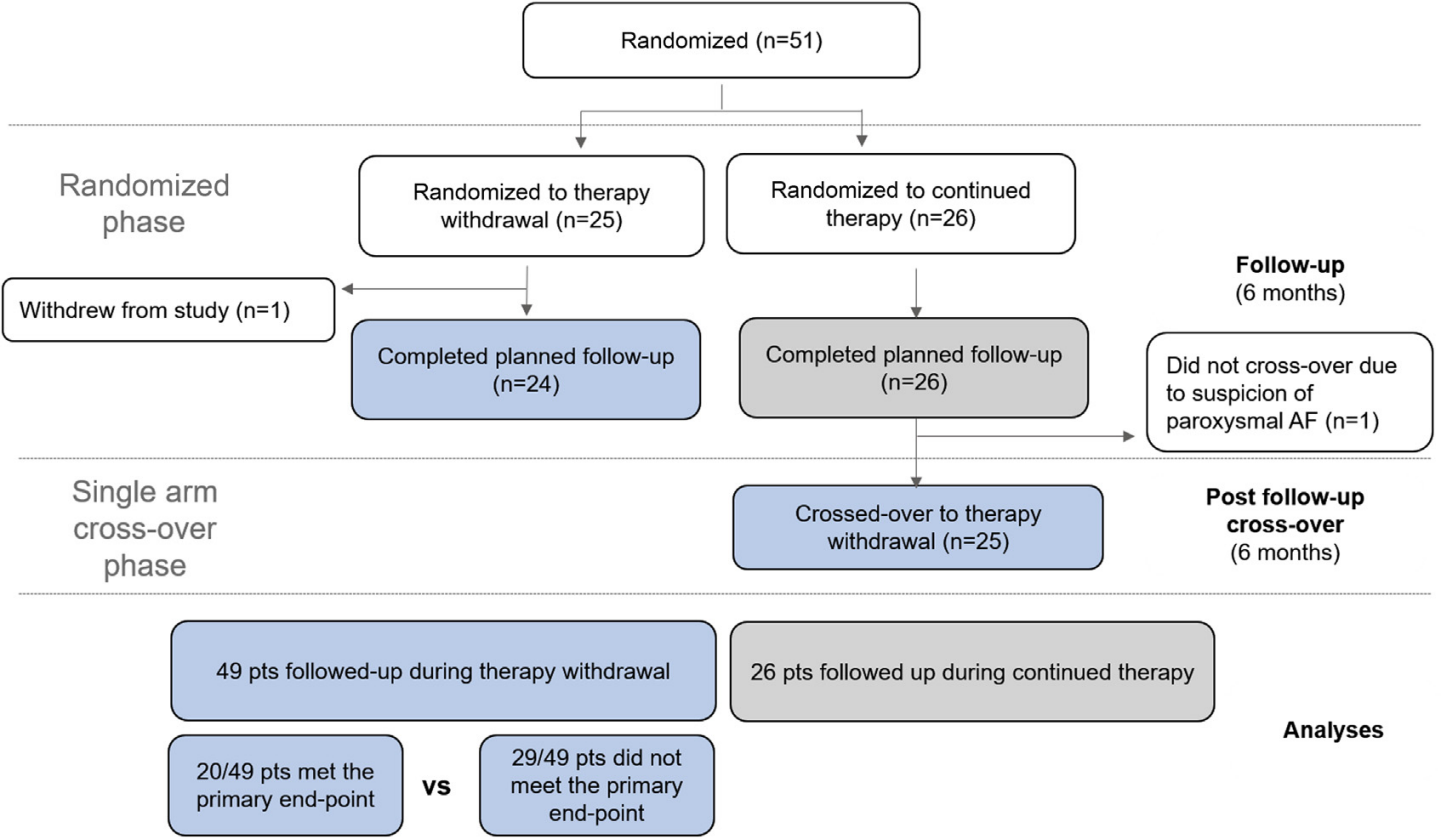
Derivation of Patients for Analyses Flow chart shows the allocation and follow-up of patients in the randomized and single-arm cross-over phases and the numbers of patients included in the current analyses.

Of 51 patients randomized, 26 were assigned to continue therapy and 25 to have therapy withdrawn. One patient randomized to the therapy withdrawal arm withdrew from the study shortly after enrolment. Another patient completed follow-up in the control arm but did not enter the single-arm cross-over phase thereafter. Therefore, heart rate data were available for 49 patients who had therapy withdrawn, 24 from the randomized phase, and 25 from the cross-over phase (Figure 1). Of those patients, 20 met the primary relapse endpoint, and 29 completed follow-up without meeting the primary endpoint. Data from 26 patients who completed follow-up in the control arm were also analyzed. No patient who continued therapy met the primary endpoint. Characteristics of patients are presented in Table 1. All patients were in sinus rhythm at baseline. Three patients developed atrial fibrillation during therapy withdrawal. One patient was immediately cardioverted without meeting the primary endpoint criteria; sinus rhythm was recorded at each study visit. Two others developed atrial fibrillation and met the primary endpoint and were therefore censored at this date.

**Table 1:** Baseline Characteristics

	Therapy Withdrawal Without Relapse (n = 29)	Therapy Withdrawal With Relapse (n = 20)	Control (n = 26)
Age, yrs	50 ± 11	59 ± 11	53 ± 13
Women	8 (28)	9 (45)	8 (31)
Heart rate, beats/min	64.6 (10.7)	68.3 (11.3)	69.9 (9.8)
Systolic BP, mm Hg	125 (10)	123 (14)	125 (12)
Diastolic BP, mm Hg	73 (10)	72 (10)	74 (8)
Weight, kg	87 (24)	84 (19)	82 (21)
Idiopathic	21 (72)	13 (65)	15 (58)
Familial	3 (10)	4 (13)	4 (15)
Environmental insult	5 (17)	4 (20)	7 (27)
*TTNtv*	6 (21)	4 (20)	4 (15)
ACE inhibitor/ARB	29 (100)	20 (100)	26 (100)
Beta-blocker	24 (82.8)	19 (95.0)	24 (92.0)
MRA	8 (27.6)	15 (75.0)	12 (46.0)
Loop diuretic	2 (6.9)	4 (20.0)	3 (12.0)
LVEF %	61 (6)	59 (6)	59 (5)
LVEDVi, ml/m^2^	78 (16)	81 (13)	80 (13)
RVEF %	58 (6)	58 (6)	59 (6)
RVEDVi, ml/m^2^	78 (18)	77 (16)	77 (17)
NT-proBNP, ng/l	50 (32–97)	111 (70–159)	75 (38–132)
Peak VO_2_, ml/kg/min∗	29 (7)	25 (5)	27 (7)
KCCQ	95 (6.0)	95 (5.0)	93 (7.5)

Values are mean ± SD, n (%), or median (interquartile range).

Characteristics taken at baseline for patients in the randomized phase and at 6 months for those having therapy withdrawn in cross-over phase.

ACE = angiotensin-converting enzyme; ARB = angiotensin receptor blocker; BP = blood pressure; BSA = body surface area; KCCQ = Kansas City Cardiomyopathy Questionnaire; LV = left ventricular; LVEDVi = left ventricular end diastolic volume indexed to body surface area; LVEF = left ventricular ejection fraction; MRA = mineralocorticoid receptor blocker; NT-pro-BNP _=_ N-terminal pro–B-type natriuretic peptide; RVEDVi = right ventricular end diastolic volume indexed to BSA; *TTNtv* = truncating genetic variants in the *TTN* gene; VO_2_ = oxygen consumption.

∗ Data were not available for n = 2 therapy withdrawal without relapse, n = therapy withdrawal with relapse, and n = 2 controls.

### Change in heart rate over follow-up

**Figure 2:**
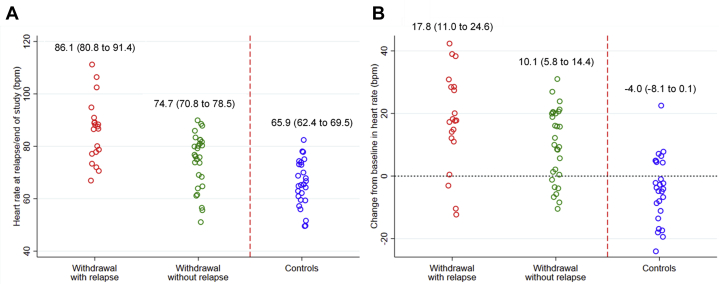
Heart Rate and Change Between Baseline and End of Study∗ According to Relapse During Follow-Up **(A)** Heart rate at relapse or the end of the study according to the occurrence of relapse. **(B)** Change in heart rate between baseline and relapse or the end of study according to the occurrence of relapse. Mean ± SD were included per set. ∗Either end of study or time of relapse.

The mean ± SD heart rate at baseline and follow-up was 64.6 ± 10.7 beats/min and 74.7 ± 10.4 beats/min for those who had therapy withdrawn but did not relapse and 68.3 ± 11.3 and 86.1 ± 11.8 beats/min for those who had therapy withdrawn and relapsed. For those who continued therapy in the control arm, the baseline and follow-up heart rates were 69.9 ± 9.8 beats/min and 65.9 ± 9.1 beats/min (Table 2, Figure 2).

**Table 2:** Changes in Heart Rates According to Relapse

	Therapy Withdrawal Without Relapse (n = 29)	Therapy Withdrawal With Relapse (n = 20)	Adjusted Mean Difference in Heart Rate at Follow-Up	p Value∗
Baseline	64.6 (10.7)	68.3 (11.3)		
4 weeks	71.8 (12.1)	76.6 (11.6)	2.7 (−3.3 to 8.6)	0.38
8 weeks	72.0 (11.0)	81.3 (10.6)	7.9 (1.9 to 13.8)	0.01
End of follow-up	74.7 (10.4)	86.1 (11.8)	10.4 (4.0 to 16.8)	0.002

Values are n (%), unless otherwise indicated.

Heart rate are shown at baseline and follow-up and changes in heart rate from baseline to follow-up and from previous visit to final visit.

∗ Using ANCOVA, adjusting for baseline heart rate

Among patients who had therapy withdrawn, the latest time points at which loop diuretics, MRAs, beta-blockers, and ACE inhibitors and ARBs were discontinued was 14, 28, 84, and 112 days, respectively. Among patients who had therapy withdrawn and relapsed, there was a persistent and steady rise in mean heart rate between baseline and 112 days (Central Illustration). Among patients who had therapy withdrawn and did not relapse, there was a less marked rise in mean heart rate between baseline and 84 days that reached a plateau thereafter (Central Illustration).

**Central Illustration:**
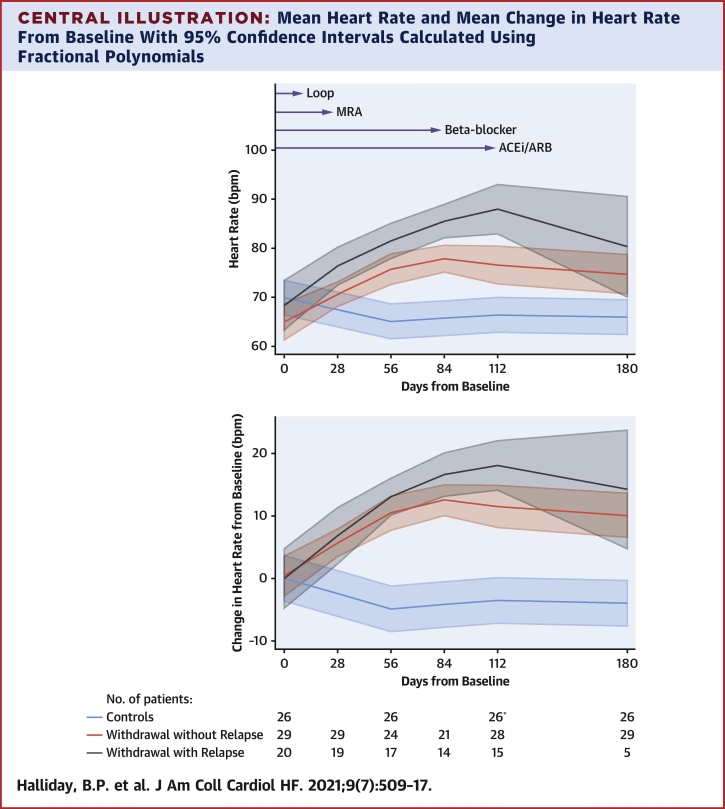
Mean Heart Rate and Mean Change in Heart Rate From Baseline With 95% Confidence Intervals Calculated Using Fractional Polynomials **Arrows** indicate the time point at which the last patient undergoing therapy withdrawal withdrew the specified medication. ∗The 16-week follow-up visit for 1 patient in the withdrawal-without-relapse group was brought forward by 4 weeks for clinical reasons. ACE = angiotensin angiotensin-converting enzyme inhibitor; ARB = angiotensin receptor blocker; Loop = loop diuretic; MRA = mineralocorticoid receptor antagonist.

After adjusting for differences in heart rate at baseline, patients who had therapy withdrawn and relapsed had a 10.4 beats/min (95% CI: 4.0 to 16.8) greater rise in heart rate over the follow-up period than patients who had therapy withdrawn and did not relapse (p = 0.002) (Table 2). The changes in heart rate between baseline and 4 weeks were not different between the groups (Table 2, Supplemental Appendix A). However, by 8 weeks, those patients who met the relapse endpoint had a 7.8 beats/min (95% CI: 1.88 to 13.82; p = 0.01) greater rise in heart rate than those who did not subsequently meet the primary endpoint (Table 2, Supplemental Appendix A).

The mean rise in heart rate after withdrawal of loop diuretics, MRAs, beta-blockers, ACE inhibitors, and ARBs was 6.7 ± 6.6 beats/min, 4.0 ± 9.8 beats/min, 14.5 ± 9.8 beats/min, and 7.0 ± 12.3 beats/min. The adjusted mean change in heart rate after withdrawal of each medication was not significantly different between patients who relapsed and those who did not (Supplemental Appendix B). There was a trend toward greater rise in mean heart rate following the withdrawal of ACE inhibitors and ARBs among patients who relapsed (5.8 beats/min; 95% CI: −0.9 to 12.6; p = 0.09).

There were no differences in heart rate changes over follow-up among carriers versus among non-carriers of truncating variants in the *TTN* gene (adjusted mean difference in heart rate at follow-up for *TTNtv* was 1.7 beats/min; 95% CI: −6.9 to 10.3; p = 0.7).

### Association between heart rate and relapse

In proportional hazard modeling, time-updated heart rate (or attained heart rate) and change in heart rate from baseline were associated with the occurrence of the primary relapse endpoint at the same visit (per 10 beats/min higher heart rate: hazard ratio [HR]: 1.92; 95% CI: 1.34–2.73; p < 0.001; per 10 beats/min change in heart rate from baseline HR: 1.99; 95% CI: 1.37–2.89; p < 0.001) (Table 3). There was no evidence that change in heart rate between the 2 most recent visits was associated with relapse (per beats/min change in heart rate from baseline HR: 1.38; 95% CI: 0.88–2.17; p = 0.16). After adjustments were made for age, log NT-proBNP concentration, and LVEF at baseline, the association between time-updated heart rate and change in heart rate from baseline and the occurrence of the relapse persisted (per 10 beats/min rise in heart rate: HR: 1.65; 95% CI: 1.10–2.57; p = 0.01; per 10 beats/min change in heart rate from baseline HR: 1.70; 95% CI: 1.12–2.57; p = 0.01). To ensure that heart rate was not a proxy for beta-blocker dose, a final sensitivity analysis was performed, adjusting for beta-blocker dose as a time-updated variable, as well as age, log NT-proBNP concentration, and LVEF at baseline. The results remained qualitatively the same (Supplemental Appendix B). A cubic spline curve demonstrated a near linear relationship between time-updated heart rate and the risk of relapse (Supplemental Appendix C).

**Table 3:** Association Between Heart Rate and Relapse

		Crude HR (95% CI)∗	p Value	Adjusted HR (95% CI)†	p Value
Model 1	Time-updated heart rate (per 10 beats/min)	1.92 (1.34 to 2.73)	<0.001	1.65 (1.10 to 2.57)	0.01
Model 2	Change in heart rate from baseline (per 10 beats/min)	1.99 (1.37 to 2.89)	<0.001	1.70 (1.12 to 2.57)	0.01
Model 3	Changes in heart rate from previous visit (per 10 beats/min)	1.38 (0.88 to 2.17)	0.16	1.22 (0.76 to 1.96)	0.42

Association among time-updated heart rate, changes in heart rate from baseline, and changes in heart rate from previous visits over days follow-up in 49 patients who underwent therapy withdrawal.

beats/min = beats per minute; LVEF = left ventricular ejection fraction; NT-proBNP = N-terminal pro–B-type natriuretic peptide

∗ Adjusted for baseline heart rate in model 2 and heart rate at previous visit in model 3.

† Adjusted for age, log NT-pro-BNP, and LVEF at baseline.

In supplemental models, time-updated heart rate (or attained heart rate), change in heart rate from baseline and change in heart rate from the previous visit were associated with the occurrence of relapse at the subsequent visit (Supplemental Appendix D). However, after adjustments were made for age, log NT-proBNP, and LVEF at baseline, these associations were not statistically significant (Supplemental Appendix D).

There was a moderate positive correlation between change in GLS and change in heart rate between baseline and follow-up (Spearman correlation coefficient = 0.37; p = 0.01). There were no significant correlations between change in LVEF (Spearman correlation coefficient = −0.18; p = 0.22), nor was a change observed in NT-proBNP (Spearman correlation coefficient = 0.04; p = 0.77) and change in heart rate (Supplemental Appendix E).

## Discussion

The present study demonstrates for the first time that attained heart rate and change in heart rate from baseline are associated with relapse among patients with recovered DCM who had therapy withdrawn. This was independent of markers of disease severity and predictors of relapse at baseline. A sensitivity analysis confirmed that heart rate was not simply a proxy for beta-blocker dose. Maintaining a lower heart rate was associated with a lower risk of relapse (Table 3). Patients who did not relapse after having therapy withdrawn had an average rise in heart rate of 10 beats/min compared to 18 beats/min for those patients who relapsed. After adjusting for differences in heart rate at baseline, the average rise in heart rate among patients who met the primary endpoint was 10 beats/min greater than those who did not relapse. The differences in heart rates between groups was observed as early as 8 weeks after the start of therapy withdrawal. In addition, the rise in mean heart rate among patients who had therapy withdrawn but did not relapse reached a plateau after 84 days, which was also the last point at which beta-blockers were withdrawn from any patient. This was in contrast to patients who met the primary endpoint, in whom average heart rate continued to rise after this point. Widening of the confidence intervals in the Central Illustration from 112 days for patients who relapsed reflects the small number of patients left in the analysis at this point.

Although there was overlap in heart rate profiles between patients who did and did not relapse, this was likely driven by use of a dichotomous measure to define the endpoint. This is of course imperfect because relapse represents adverse cardiac remodeling, which is a continuous phenomenon. Some patients who had therapy withdrawn and did not meet the primary relapse endpoint still had evidence of adverse remodeling associated with a rise in heart rate. Longer follow-up is likely to have identified further cases of relapse. The overlap between the 2 groups represents the spectrum of remodeling across these patients. Calculating the sensitivity and specificity of a measure to detect a dichotomized definition of relapse, therefore, has obvious limitations and is likely to underestimate its usefulness.

Although the high rate of relapse in patients with recovered DCM observed in TRED-HF suggests that most patients should continue to take disease-modifying pharmacological therapy (3); some patients and clinicians may still decide to reduce therapy in specific situations. Women with a previous diagnosis of peripartum cardiomyopathy may want to try to become pregnant again and elect to stop taking renin-angiotensin-aldosterone blockers (12,13). Where an obvious cause for DCM, such as persistent tachycardia or viral myocarditis, has resolved, withdrawing treatment may be considered logical. In other situations, patients may perceive the risk of side effects related to medications and the associated reduction in quality of life to be greater than the risk of relapse. This is, after all, an individual decision made by the patient. Patients and clinicians may also choose to withdraw specific medications, such as MRAs or to reduce the dose of medications rather than abandon all treatments. Given the high-risk of relapse, it is crucial that we have effective non-invasive measurements to monitor patients, identify early signs of relapse, and reinstitute therapy before clinical decompensation occurs.

Our data suggest that changes in heart rates may be used as a non-invasive marker of relapse to guide the use of cardiac imaging and testing of natriuretic peptide. Follow-up with regular CMR, as used in this trial, is rarely feasible in clinical practice. Incorporation of a strategy informed by heart rate may facilitate the tailored and rapid reintroduction of pharmacological therapy and prevent clinical relapse. Advances in digital health care technology allow heart rate to be monitored using wearable or hand-held devices. The appearance of differences in heart rates between groups as early as 8 weeks following the start of therapy withdrawal raises the possibility of using heart-rate monitoring to predict future relapse. Altogether, until more robust evidence is available, a multiparametric approach appears advisable, with frequent assessment of heart rate and symptoms supported by less frequent assessment by imaging and biomarkers. Based on these data, a heart rate rise of >10 beats/min from baseline may act as a prompt for expedited imaging investigation. Validation of specific heart rate cutoffs is required and could be incorporated into future clinical trials investigating the management of patients with DCM and improved cardiac function.

It is uncertain whether the rise in heart rate observed in this study was the cause or the consequence of worsening cardiac function. Although the withdrawal of each class of medication was associated with an overall rise in mean heart rate, the magnitude of this was greatest with beta-blockers. The rise in heart rate seen following the withdrawal of non-rate-slowing medications, suggests that it may be a consequence of decompensation and increasing myocardial workload. However, we would advise against over-interpretation of these results, given the modest number of patients taking loop diuretic agents and MRAs. It should also be borne in mind that medications were withdrawn sequentially over a relatively short period of time and that the change in heart rate observed during the withdrawal of ACE inhibitors and ARBs might have been influenced by the preceding withdrawal of beta-blockers. As demonstrated in the primary publication, those patients who relapsed had markers of more severe disease at baseline with higher concentrations of natriuretic peptides and also were older (3). It is also important to emphasize that most patients in this study had idiopathic or familial disease and that dedicated studies focusing on specific causes such as peripartum cardiomyopathy are needed.

If the rise in heart rate is an important cause for relapse, then maintaining rate-slowing medications at a dose sufficient to control heart rate may prevent relapses, allowing other medications to be reduced. Adrenergic receptor blockade may be just one means of reducing heart rate. For patients in sinus rhythm, ivabradine also improves ventricular function and clinical outcomes (8,14). Further research in larger numbers of patients is required before such a recommendation could be endorsed by guidelines. The present authors also emphasize that these results should not be applied to patients with atrial fibrillation where the relationship between heart rate and prognosis is different and where tight heart control may be detrimental and also patients with ischemic heart disease where reversibility of disease is even more doubtful and therapy withdrawal is likely to be riskier (15,16).

## Conclusions

Among patients among patients with recovered DCM who had disease-modifying pharmacological therapy for heart failure withdrawn, attained heart rate and change in heart rate from baseline were associated with relapse. A strategy that combines frequent measurements of heart rate with less frequent imaging assessment may be a safe and effective way of monitoring treatment withdrawal or tapering, for patients where this is performed out of necessity, based on clinical circumstances or patient choice. A lower heart rate was associated with a lower risk of relapse. Whether heart rate control is a key determinant for maintaining remission of DCM deserves further investigation.

Perspectives **COMPETENCY IN MEDICAL KNOWLEDGE:** A rise in heart rate is associated with adverse remodeling and relapse among patients with recovered dilated cardiomyopathy whose medication is withdrawn. Combining heart rate measurement with imaging and natriuretic peptide measurement may be an effective way of monitoring patients who elect to have medications withdrawn under specific circumstances. A heart rate rise of >10 beats/min may be used as a guide to prompt further investigation.

**TRANSLATIONAL OUTLOOK:** It remains unclear whether change in heart rate is a marker or mediator of adverse remodeling in patients with recovered dilated cardiomyopathy. Whether heart rate suppression is the key to maintaining remission deserves further investigation.

## Funding Support and Author Disclosures

The TRED-HF study was an investigator-led trial sponsored by Royal Brompton and Harefield National Health Service Trust. The study was funded by a Clinical Research Training Fellowship from the British Heart Foundation (FS/15/29/31492) awarded to Dr Halliday and Prasad. Additional support was received from the Alexander Jansons Foundation; the Cardiovascular Research Centre; and National Institute for Health Research (NIHR) Biomedical Research Unit at Royal Brompton Hospital; the NIHR Imperial College Biomedical Research Centre; and by grants from Rosetrees Trust, awarded to Dr Prasad. Dr Halliday is supported by a Clinical Lecturer Starter Grant from the Academy of Medical Sciences (SGL021∖1025). Prof John Cleland received a Centre of Research Excellence award from the British Heart Foundation (RE/18/6/34217). Prof Pennell has received research support from Siemens; and speaker fees from Chiesi and Bayer; is a consultant for ApoPharma and Chiesi; and is a director and owns stock in Cardiovascular Imaging Solutions. Prof Cowie has received personal fees from Servier and Novartis, outside the submitted work. Prof Cleland has received personal fees from Abbott, and grants and personal fees from Amgen, Bayer, Medtronic, Novartis, Pharmacosmos, Vifor, Bristol Myers Squibb, and Servier, outside the submitted work. All other authors have reported that they have no relationships relevant to the contents of this paper to disclose.
