# Supplementary material for: Heart Rate as a Marker of Relapse During Withdrawal of Therapy in Recovered Dilated Cardiomyopathy
Source: JACC Heart Fail. 2021 Jul;9(7):509–17. doi: 10.1016/j.jchf.2021.03.010 (PMC8259664; doi:10.1016/j.jchf.2021.03.010)
Supplement: Supplemental Data [file mmc1.docx]

**Appendix**

**Appendix A: Change in heart rate between baseline and 4 weeks and baseline and 8 weeks stratified by the occurrence of relapse**


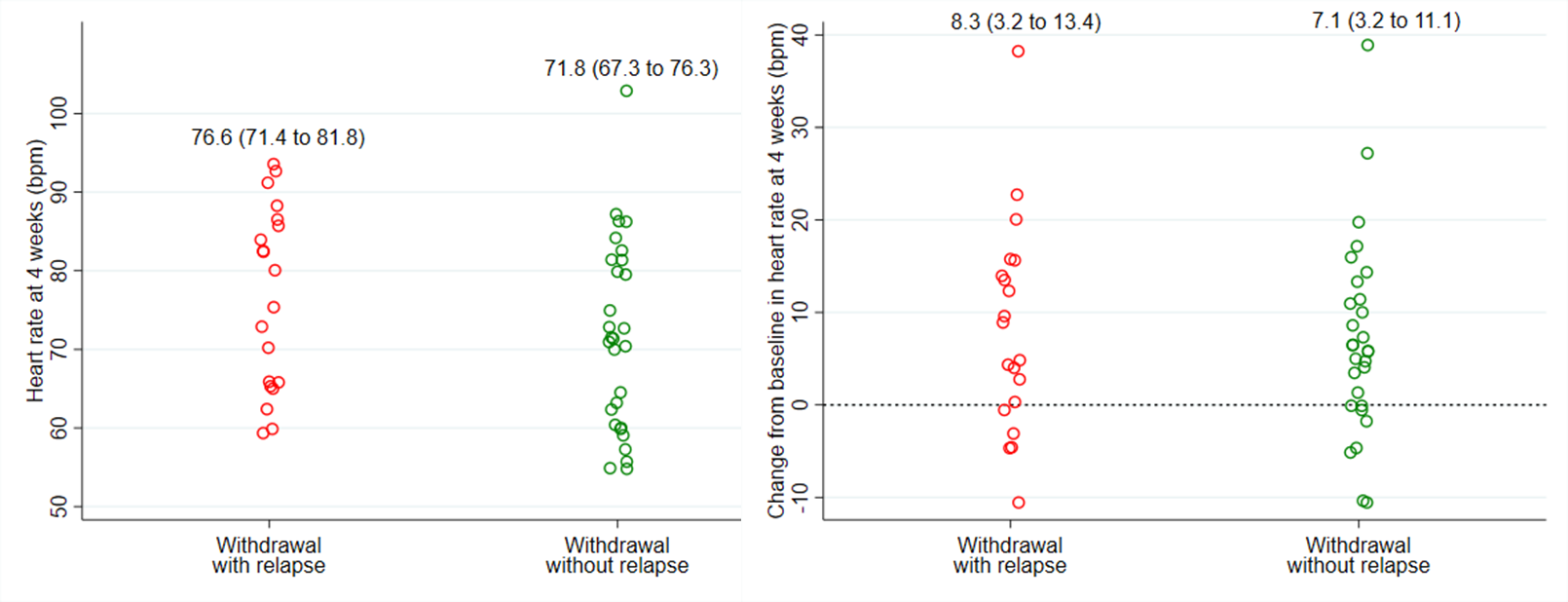


**
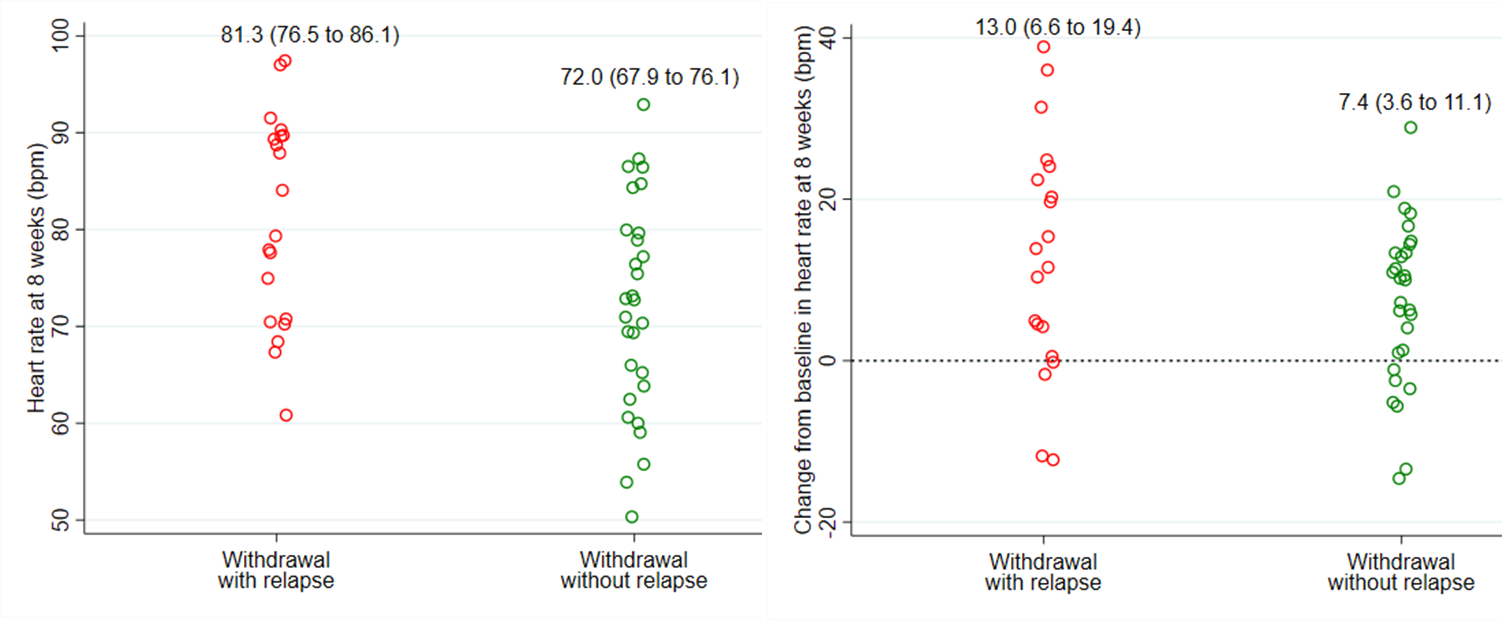
**

**Appendix B: Change in heart rate according to relapse and withdrawal of heart failure therapy**

Mean heart rate and mean change in heart rate immediately before and after the withdrawal of each individual medication and stratified by relapse status

|  | **All therapy withdrawal patients** | | **Therapy withdrawal without relapse** | | **Therapy withdrawal with relapse** | | **Adjusted mean difference in heart rate**  (95% CI) | **P value^a^** |
| --- | --- | --- | --- | --- | --- | --- | --- | --- |
|  | Mean (SD) | Mean change (SD) | Mean (SD) | Mean change (SD) | Mean (SD) | Mean change (SD) |  |  |
| **Beta-blocker (N=40)** | | | | | | | | |
| Last full dose | 66.2 (9.1) | 14.5 (9.8) | 64.7 (10.0) | 13.5 (9.1) | 68.4 (7.4) | 16.0 (10.8) | 3.7 (-2.7, 10.1) | 0.25 |
| Withdrawal | 80.7 (11.5) |  | 78.2 (11.5) |  | 84.4 (10.8) |  |  |  |
| **ACEi/ARB (N=46)** | | | | | | | | |
| Last full dose | 71.9 (13.0) | 7.0 (12.3) | 68.6 (13.2) | 6.8 (10.9) | 77.6 (10.9) | 7.3 (14.9) | 5.8 (-0.9, 12.6) | 0.09 |
| Withdrawal | 78.9 (12.2) |  | 75.3 (11.2) |  | 84.9 (11.7) |  |  |  |
| **MRA (N=23)** | | | | | | | | |
| Last full dose | 68.9 (10.8) | 4.0 (11.2) | 65.3 (7.8) | 4.5 (7.7) | 70.9 (11.8) | 3.7 (12.9) | 2.3 (-7.1, 11.7) | 0.61 |
| Withdrawal | 72.9 (10.8) |  | 69.8 (10.4) |  | 74.5 (11.0) |  |  |  |
| **Loop diuretics (N=6)** | | | | | | | | |
| Last full dose | 67.5 (13.5) | 6.7 (6.6) | 59.0 (5.7) | 4.5 (6.4) | 71.8 (14.9) | 7.8 (7.4) | 6.2 (-17.2, 29.7) | 0.46 |
| Withdrawal | 74.2 13.5) |  | 63.5 12.0) |  | 79.5 12.0) |  |  |  |

^a^ Using ANCOVA adjusting for baseline heart rate

ACE – angiotensin converting enzyme; ARB: angiotensin receptor blocker; MRA: mineralocorticoid receptor blocker

**Appendix C: Sensitivity analysis of models 1, 2 and 3, adjusting for beta-blocker dose, age, log NT-pro-BNP and LVEF**

|  |  | **Adjusted HR (95% CI)** | **P value** |
| --- | --- | --- | --- |
| **Model 1** | **Heart rate (per 10bpm)** | 1.65 (1.10, 2.47) | 0.02 |
| **Model 2** | **Change in heart rate from baseline (per bpm)** | 1.05 (1.01, 1.10) | 0.01 |
| **Model 3** | **Change in heart rate from previous visit (per bpm)** | 1.02 (0.97, 1.07) | 0.43 |

* where beta-blocker dose is included as a time-updated covariate

Bpm – beats per minute; CI – confidence intervals


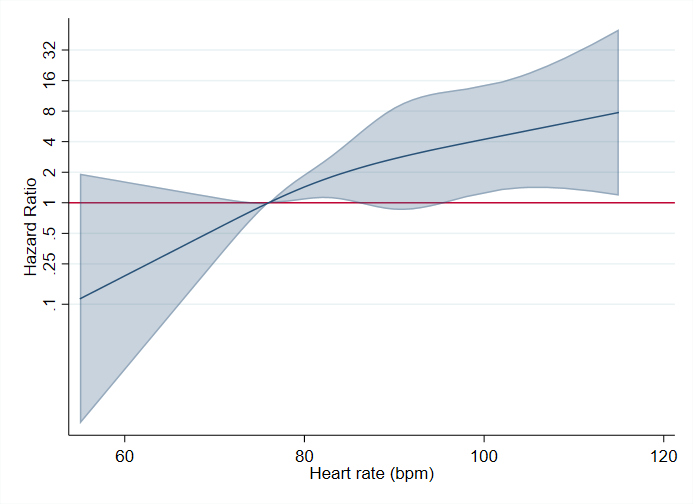
**Appendix D: Estimated hazard ratio for relapse by resting time-updated heart rate compared to mean heart rate**

Mean time-updated heart rate used as reference.

**Appendix E: Association between 1) time-updated heart rate, 2) change in heart rate from baseline and 3) change in heart rate from previous visit over days follow up in 49 patients who underwent therapy withdrawal.***

|  |  | **Unadjusted hazard ratio** (95% CI)** | **P**  **value** | **Adjusted hazard ratio *** (95% CI)** | **P value** |
| --- | --- | --- | --- | --- | --- |
| **Model 1** | **Time-updated heart rate (per 10bpm)** | 1.58 (1.10, 2.52) | 0.01 | 1.32 (0.87, 1.98) | 0.19 |
| **Model 2** | **Change in heart rate from baseline (per bpm)** | 1.05 (1.01, 1.10) | 0.02 | 1.04 (0.99, 1.09) | 0.11 |
| **Model 3** | **Change in heart rate from previous visit (per bpm)** | 1.06 (1.01, 1.11) | 0.02 | 1.04 (1.00, 1.09) | 0.07 |

* Investigating the association between heart rate at previous visit and relapse

** Adjusted for baseline heart rate in model 2 and heart rate at previous visit in model 3

*** Adjusted for age, log NT-pro-BNP and LVEF at baseline

Bpm – beats per minute; CI – confidence intervals

**Appendix F: Correlation between change in heart rate and change in measures of left ventricular systolic function and natriuretic peptide concentrations.**

1. The association between change in heart rate (bpm) and change in LVEF (%) from baseline to relapse/end of study


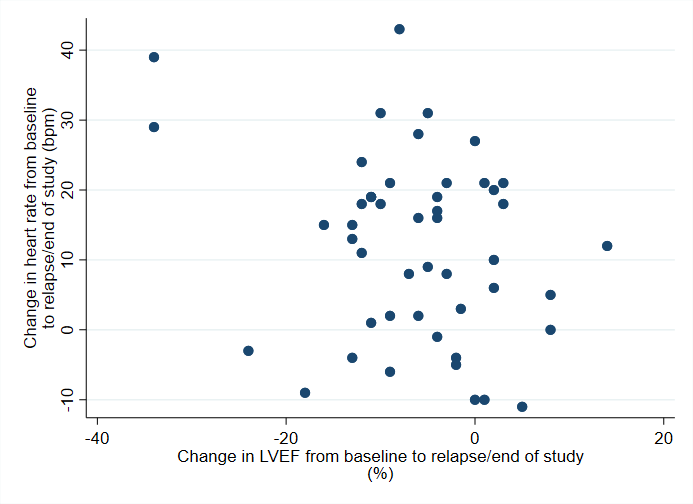


r = -0.18

1. The association between change in heart rate (bpm) and change in LV GLS from baseline to relapse/end of study.


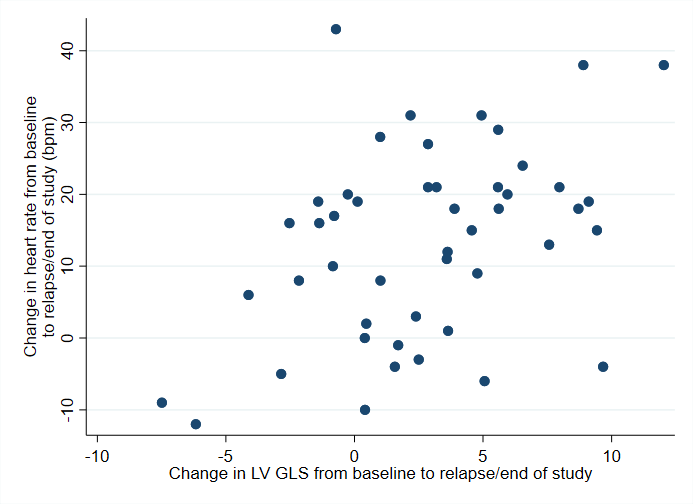


r = 0.37

1. The association between change in heart rate (bpm) and change in NT-pro BNP from baseline to relapse/end of study.


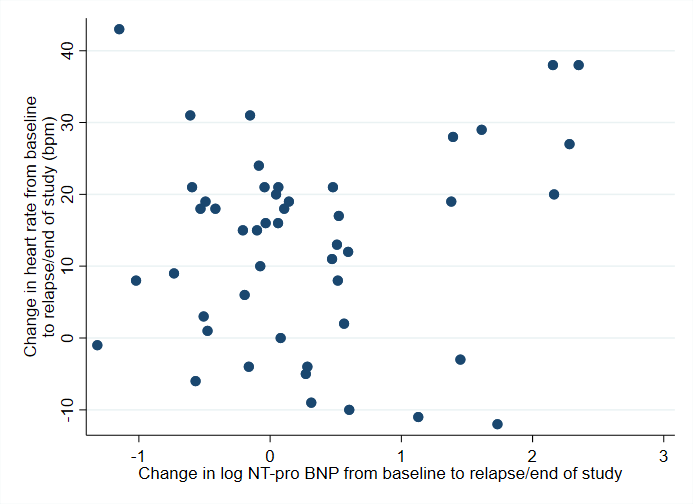


r = 0.04
